# Neural decoding of Aristotle tactile illusion using deep learning-based fMRI classification

**DOI:** 10.3389/fnins.2025.1606801

**Published:** 2025-06-19

**Authors:** Eunji Lee, Ji-Hyun Kim, Jaeseok Park, Sung-Phil Kim, Taehoon Shin

**Affiliations:** ^1^Department of Mechanical and Biomedical Engineering, Ewha W. University, Seoul, Republic of Korea; ^2^Graduate Program in Smart Factory, Ewha W. University, Seoul, Republic of Korea; ^3^Department of Cognitive and Psychological Sciences, Brown University, Providence, RI, United States; ^4^Department of Biomedical Engineering, Sungkyunkwan University, Suwon, Republic of Korea; ^5^Department of Intelligent Precision Healthcare Convergence, Sungkyunkwan University, Suwon, Republic of Korea; ^6^Department of Biomedical Engineering, Ulsan National Institute of Science and Technology, Ulsan, Republic of Korea; ^7^Department of Artificial Intelligence and Software, Ewha W. University, Seoul, Republic of Korea

**Keywords:** somatosensory, tactile illusion, fMRI, deep learning, brain mapping

## Abstract

**Introduction:**

Aristotle illusion is a well-known tactile illusion which causes the perception of one object as two. EEG analysis was employed to investigate the neural correlates of Aristotle illusion, yet was limited due to low spatial resolution of EEG. This study aimed to identify brain regions involved in the Aristotle illusion using functional magnetic resonance imaging (fMRI) and deep learning-based analysis of fMRI data.

**Methods:**

While three types of tactile stimuli (Aristotle, Reverse, Asynchronous) were applied to thirty participants’ fingers, we collected fMRI data, and recorded the number of stimuli each participant perceived. Four convolutional neural network (CNN) models were trained for perception-based classification tasks (the occurrence of Aristotle illusion vs. Reverse illusion, the occurrence vs. absence of Reverse illusion), and stimulus-based classification tasks (Aristotle vs. Reverse, Reverse vs. Asynchronous, and Aristotle vs. Asynchronous).

**Results:**

Simple fully convolution network (SFCN) achieved the highest classification accuracy of 68.4% for the occurrence of Aristotle illusion vs. Reverse illusion, and 80.1% for the occurrence vs. absence of Reverse illusion. For stimulus-based classification tasks, all CNN models yielded accuracies around 50% failing to distinguish among the three types of applied stimuli. Gradient-weighted class activation mapping (Grad-CAM) analysis revealed salient brain regions-of-interest (ROIs) for the perception-based classification tasks, including the somatosensory cortex and parietal regions.

**Discussion:**

Our findings demonstrate that perception-driven neural responses are classifiable using fMRI-based CNN models. Saliency analysis of the trained CNNs reveals the involvement of the somatosensory cortex and parietal regions in making classification decisions, consistent with previous research. Other salient ROIs include orbitofrontal cortex, middle temporal pole, supplementary motor area, and middle cingulate cortex.

## 1 Introduction

The ability to perceive and interpret sensations is fundamental to human interaction with the environment. In some instances, we perceive external stimuli in ways that differ from reality. Such perceptual illusions occur when a stimulus delivered under specific conditions elicits a different conscious experience due to changes in those conditions (Hayward, 2008). By examining how the brain produces illusory perceptions, researchers can gain insights into the neural mechanisms underlying sensory integration and perception.

Although perceptual illusions have been documented across various sensory modalities, most research has focused on visual illusions (Robinson, 2013; Keil, 2020; Manassi and Whitney, 2022). This emphasis has yielded a detailed understanding of the neural basis of visual perception; however, comparatively less is known about the neural basis of tactile illusions. Among tactile illusions, the Aristotle illusion is one of the well-known perceptual phenomena. The Aristotle illusion occurs when a person crosses two fingers and touches a single object on the medial side of the crossed fingers, leading to the perception of one object as two. Conversely, if two objects are touched on the external sides of the crossed fingers, they are perceived as one (Benedetti, 1985). This phenomenon may result from the neural integration of cutaneous tactile inputs with proprioceptive information regarding the atypical (crossed) positioning of the stimulated skin locations.

Despite its simplicity, the Aristotle illusion offers a unique opportunity to study how the brain resolves conflicting tactile and proprioceptive inputs to support perceptual decision-making. Specifically, it offers a tractable framework for exploring how the brain selects among competing perceptual hypotheses under an illusory condition. This process involves not only integrating somatotopically organized tactile signals with proprioceptive representations but also evaluating conflicting sensory cues to construct a coherent percept. Identifying the cortical regions supporting this resolution process offers insight into how the brain stabilizes perceptual experience in the presence of contradictory input.

A previous study employed EEG and source localization to investigate the neural correlates of the Aristotle illusion (Bufalari et al., 2014). Researchers found that the P200 component from the posterior parietal cortex (PPC) was stronger when participants did not perceive the illusion compared to when they did. However, the low spatial resolution of EEG poses challenges for detecting neural activity related to the Aristotle illusion from deep brain regions. Indeed, previous research has implicated several subcortical and middle regions in tactile information, such as the thalamus, cingulate cortex, insular cortex and frontal cortex (Allen et al., 2016). Neuroimaging studies on proprio-tactile illusion have also highlighted deep brain regions, including the supplementary motor area (SMA) and thalamus (Kavounoudias et al., 2008). Given these findings, functional magnetic resonance imaging (fMRI), with its high spatial resolution and ability to examine neural responses in deep brain regions, is well suited for analyzing whole-brain activity patterns associated with the Aristotle illusion.

Previous fMRI studies on tactile illusions have revealed several brain regions involved in the illusory processing of tactile information. In the cutaneous rabbit illusion, where rapid stimulation at distinct arm points creates the sensation of intermediate “hopping” tactile stimuli, increased blood oxygen level-dependent (BOLD) signals were observed in the primary somatosensory cortex (S1) at unstimulated skin locations as well as in premotor and prefrontal regions (Blankenburg et al., 2006). The velvet hand illusion, which induces the sensation of a velvety texture using a grid of wires between the hands, showed enhanced activation in S1 and increased connectivity with somatosensory-related regions (Rajaei et al., 2018). Yet, no fMRI study to date has investigated the neural correlates of the Aristotle illusion.

In previous fMRI studies, researchers observed the neural correlates of tactile illusion through univariate analysis (Friston et al., 1994). This analytical approach relies on correlations between individual BOLD signals and the predicted hemodynamic response function (HRF) by stimulus designs via the general linear model applied to each individual voxel. However, neural responses to stimuli may be better explained in a high-dimensional space. The functional relationships between neural responses and stimuli may be nonlinear, and the shape of the HRF can deviate from the conventional canonical form across different participants or individual voxels (Aguirre et al., 1998; Chen et al., 2023). Additionally, recent findings have raised concerns about the effectiveness of univariate analysis in predicting individual differences (Kragel et al., 2021). One approach to overcoming these limitations is the use of multi-variate decoding analyses based on machine learning techniques (Kriegeskorte et al., 2006; Norman et al., 2006). In recent years, researchers have successfully decoded neural responses to various stimuli using multivoxel pattern analysis (MVPA) in the tactile perception domain (Kim et al., 2017; Kim et al., 2019). However, traditional MVPA techniques require feature selection and extraction processes and are limited when applied to high-dimensional raw data.

Deep learning has emerged as a powerful technique for medical image analysis following the great success of convolutional neural networks (CNNs) in the natural image domain. CNNs enable fully automated extraction of important image features and facilitate end-to-end prediction without the need for manual feature engineering. Consequently, CNNs have been applied to fMRI data for the diagnosis of Alzheimer’s disease, autism, and schizophrenia (Sarraf and Tofighi, 2016; Meszlényi et al., 2017; Yin et al., 2022). Beyond disease diagnosis, fMRI-based CNN models have also been developed for classifying brain task states, including visual brain states, sensorimotor task states, emotional states, and others (Zhang et al., 2023; Vu et al., 2020; Tchibozo et al., 2022; Wang et al., 2020). Another significant advancement in medical deep learning has been the development of model interpretation techniques to counter the “black-box” nature inherent in artificial neural networks. Class activation mapping (CAM) and its various extensions are among the most established techniques for visualizing the decision-making processes of CNNs (Selvaraju et al., 2017). Gradient-weighted CAM (Grad-CAM) family has been employed in diverse brain MRI applications, including the classification of multiple sclerosis, prediction of seizure onset zones, detection of brain tumors, and categorization of degenerative neurological diseases (Zhang et al., 2021; Luckett et al., 2022; Mahesh et al., 2024; Song et al., 2024).

This study investigated deep learning methods that directly analyze whole-brain fMRI data related to the Aristotle illusion in an end-to-end manner. While one or two tactile stimuli were applied to participants’ fingers, we collected associated fMRI data and recorded the number of stimuli each participant perceived. We developed CNN models which classify these fMRI data according to the type of applied stimulus and the number of perceived stimuli. We then applied Grad-CAM to identify and visualize brain regions considered important for the classification decisions made by the trained CNNs. We assumed that the regions highlighted by Grad-CAM contribute to classification performance by exhibiting distinct activation patterns depending on the stimulus type or the perceptual experience. Based on previous studies, we hypothesized that somatosensory and parietal regions would show significant differences in classification accuracy across illusion conditions. Furthermore, we aimed to identify deep brain regions that were not detected in earlier studies of Aristotle illusion. These findings advance our understanding of the mechanisms underlying the Aristotle illusion, specifically regarding how the brain constructs tactile percepts in illusory contexts. To the best of our knowledge, this was the first study to apply deep learning methods to examine brain activation patterns associated with tactile stimulation using novel fMRI data recorded concurrently during stimulus presentation.

## 2 Materials and methods

### 2.1 Participants

Thirty participants (15 females; mean age ± standard deviation: 24.6 ± 2.4) with no contraindications for MRI and no history of neurological disorders were included in this study. Only right-handed participants were recruited to control for handedness effects. The study was approved by the ethics committee of the Ulsan National Institute of Science and Technology (UNISTIRB-17-20-A). All participants were informed of the study objectives and experimental procedures and voluntarily provided written informed consent.

### 2.2 Tactile stimuli

**Figure 1:**
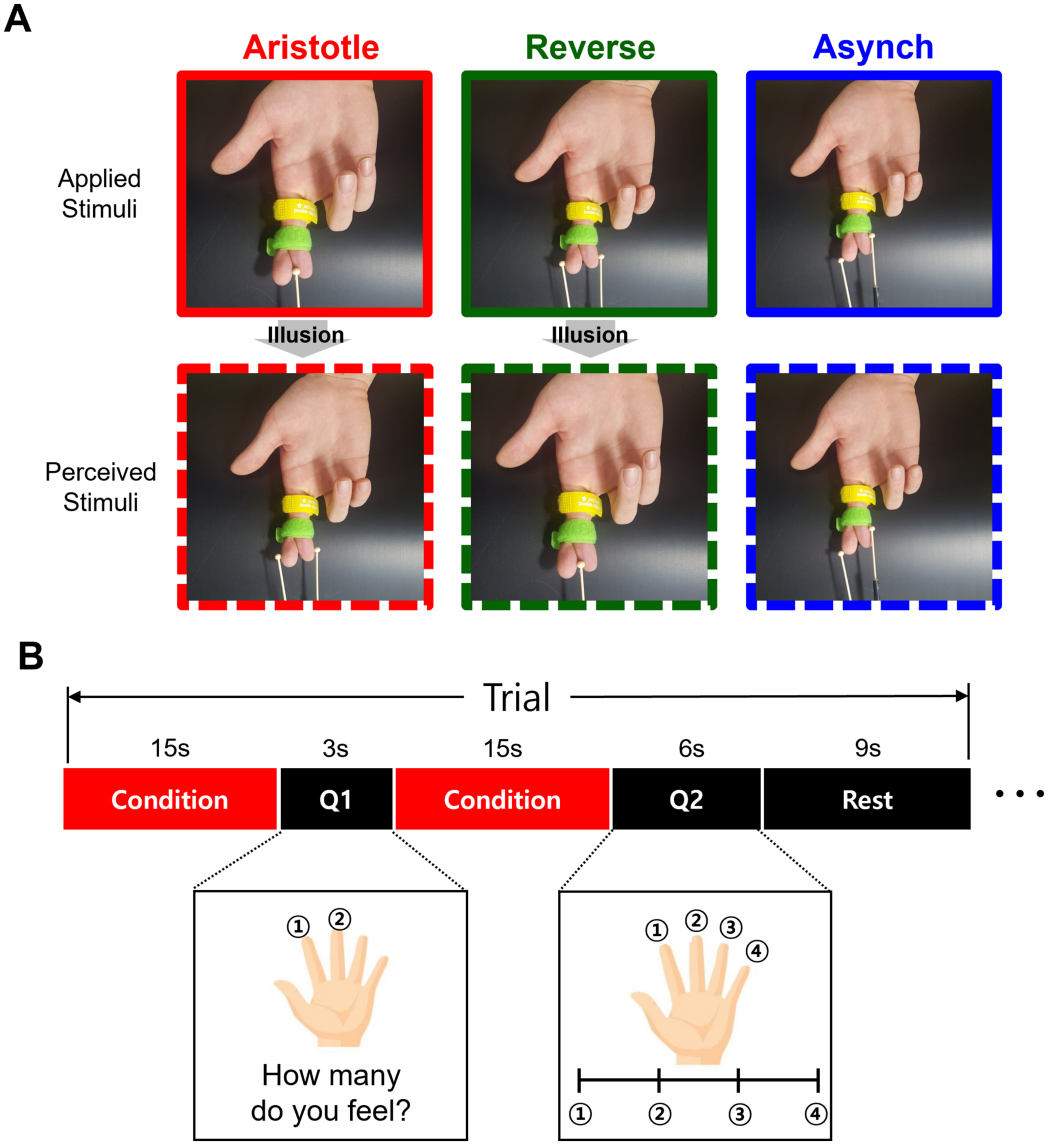
Tactile stimulation and experimental paradigm. **(A)** For the Aristotle stimuli, only one stimulus is administered, but participants perceive two objects. For the Reverse stimuli, two stimuli are administered, but only one object is perceived. For the Asynchronous (control) stimuli, two stimuli are administered while the fingers are crossed, and participants perceive two objects without experiencing the illusion. **(B)** The experimental procedure consisted of two questions. The first question asked how many stimuli were perceived, allowing determination of whether the illusion occurred. The second question asked about the perceived distance between the stimuli. In each trial, the tactile stimulus was presented for 15 s, after which participants answered the first question. In the next step, the same stimulus was presented again for another 15 s, followed by the second question. The three types of stimuli—Aristotle, Reverse, and Asynchronous—were randomly assigned. Each stimulus was repeated for five trials, resulting in 15 trials per session, with each participant completing two sessions.

We adopted the design of tactile stimuli from the previous study in which the Aristotle illusion was observed (Bufalari et al., 2014). During the experiment, a Velcro tape held the participants’ right-hand fingers crossed to prevent discomfort or involuntary muscle movement that might arise from actively maintaining this unnatural posture (Figure 1A). The experimenter administered tactile stimulation by moving wooden balls (6 mm in diameter) attached to a stick along the distal phalanges of the crossed fingers. The experimenter was trained to maintain a consistent frequency of approximately 1.5 cycles per second, applying a controlled force across three different tactile stimulation conditions (see below). Only the wooden balls made contact with the participants’ skin. Three distinct stimulation conditions were presented as follows. In the Aristotle condition, a single ball stimulus was applied to the middle of the crossed fingers (right index and middle), potentially leading to the illusory perception of two stimuli. In the reverse condition, two stimuli were synchronously applied to both lateral ends of the crossed fingers, possibly inducing the illusory perception of a single stimulus. In addition to these mismatch conditions between actual and perceived stimuli, the Asynchronous condition was included, in which two asynchronously delivered stimuli were veridically perceived as two separate contacts. In both the Aristotle and Reverse conditions, the corresponding areas of the crossed fingers were touched simultaneously. In contrast, during the Asynchronous condition, the corresponding finger areas were stimulated at different time intervals (Figure 1A).

### 2.3 fMRI experiment

We applied the experimental procedure from the previous study (Bufalari et al., 2014) with modifications tailored to the fMRI experiments. Prior to the fMRI session, participants were visually shown the tactile stimuli and informed that they might perceive either one or two balls at the central or lateral areas of their crossed right middle and index fingers. In addition, the experimenter informed participants that two questions would be asked in each trial. After the initial stimulation, the first question assessed the number of stimuli perceived, which determined whether an illusion had occurred. Participants were instructed to press the ‘1’ button with their left index finger if they felt a single stimulus, and the ‘2’ button with their left middle finger if they felt two stimuli. Following the presentation of an identical second stimulus, the second question inquired about the perceived spatial distance between the stimuli. Participants were instructed to respond with ‘1’ using their left index finger if they felt a single stimulus or were unable to distinguish a spatial difference. They responded with ‘4’ using their left little finger if they perceived the stimuli as being separated by the full width of their crossed fingers. If the stimuli felt farther apart than button ‘1’ but less than the finger width, they were instructed to respond with ‘2’ with their left middle finger; if wider than ‘2’ but narrower than ‘4,’ they were to respond with ‘3’ with their left ring finger. Participants were asked to respond as accurately and quickly as possible.

During the fMRI experiment, three types of tactile stimuli (Aristotle, Reverse, and Asynchronous) were presented, with each type administered in five trials within a single session (Figure 1B). Each participant completed two fMRI sessions, performing a total of 30 trials (10 repetitions of each tactile stimulus). The sequence of the 30 trials was fully randomized for each participant. Only one type of tactile stimulus was presented within each trial. In each trial, the experimenter presented a single type of tactile stimulus for 15 s, during which the word “Stimulating” appeared on the screen. Afterward, participants responded to the first question, which was displayed for 3 s. Responses were recorded using a button box held in the left hand. Following the first question, the experimenter presented the same type of tactile stimulus again for another 15 s. Subsequently, the second question was displayed for 6 s. Each trial lasted 48 s and was followed by a 9-s resting period. Each session lasted 720 s, resulting in a total experimental time of 1,440 s per participant.

### 2.4 MRI acquisition and preprocessing

MRI scanning was performed using a 3 T scanner (Magnetom TrioTim, Siemens, Germany) equipped with a 64-channel head coil at the Center for Neuroscience Imaging Research in Suwon, Republic of Korea. Functional images were acquired using a slice-accelerated multiband gradient-echo-based echo planar imaging (EPI) sequence with T2*-weighted blood oxygenation level-dependent (BOLD) contrast. Functional images covering the entire brain were obtained (48 slices; repetition time (TR) = 3 s; echo time (TE) = 30 ms; flip angle = 90°; Field of view (FOV) = 192 mm; slice thickness = 3 mm; voxel size = 2.0 × 2.0 × 3.0 ${\mathrm{mm}}^{3}$ ). High-resolution anatomical images were also obtained using a T1-weighted 3D MPRAGE sequence (TR = 2,300 ms; TE = 2.28 ms; flip angle = 8°; FOV = 256 mm; voxel size = 1.0 × 1.0 × 1.0 ${\mathrm{mm}}^{3}$ ). Functional images were preprocessed using SPM12 software (Wellcome Department of Imaging Neuroscience, London, UK) with standard procedures, including slice-timing correction, re-alignment, co-registration, segmentation, and spatial normalization to the Montreal Neurological Institute (MNI) template.

### 2.5 Behavioral data analysis

We categorized trials as either illusion trial s or non-illusion trials based on how participants perceived the stimuli under different conditions. In the Aristotle stimulation condition, a trial was classified as an illusion trial if participants reported perceiving two stimuli. In the Reverse and Asynchronous conditions, a trial was categorized as an illusion trial if participants reported perceiving one stimulus. The illusion rate was defined as the proportion of illusion trials among all trials in which the participant provided a response. Statistical analysis is described in Section 2.8.

### 2.6 fMRI classification tasks

The fMRI data acquired during the application of tactile stimuli were analyzed using deep learning-based classification. A total of five classification tasks were formulated based on two criteria, as described below. First, based on the type of stimulus *applied*, binary classification was performed for each of the following pairs of stimulus categories: Aristotle (*n* = 596) vs. Reverse (*n* = 594), Reverse vs. Asynchronous (*n* = 590), and Aristotle vs. Asynchronous. Second, based on the number of stimuli *felt* by participants, binary classification was conducted for the occurrence of the Aristotle illusion (*n* = 544) vs. Reverse illusion (*n* = 212), corresponding to experiences of two vs. one stimulus, respectively, as well as for the occurrence (*n* = 212) vs. absence (*n* = 382) of the Reverse illusion, corresponding to experiences of one vs. two stimuli, respectively. Other potential classification tasks, including the absence of the Aristotle illusion (52 cases), were excluded due to the insufficient sample size, which would lead to severe class imbalance.

### 2.7 CNN learning and analysis

**Figure 2:**
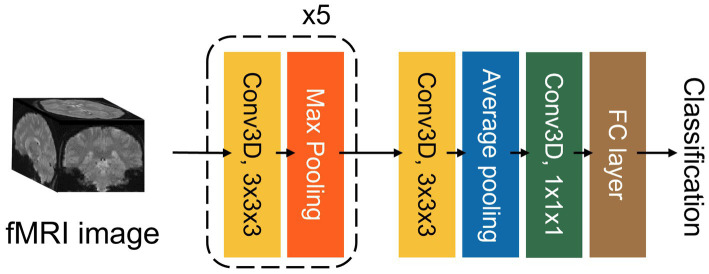
Architecture of the SFCN. The SFCN is a lightweight 3D CNN model consisting of six blocks, each composed of a convolution layer followed by a maximum or average pooling for feature extraction, and a final block comprising a convolution layer followed by a fully connected layer for decision-making.

We employed and compared four CNN models for fMRI classification: ResNet10, ResNet18, DenseNet121, and a Simple Fully Convolution Network (SFCN). ResNet is a well-established CNN architecture characterized by residual connections that improve the gradient-based optimization of model parameters (He et al., 2016). DenseNet is distinguished by its layer-wise concatenation, which enhances representational capacity and was selected as a representative of large-scale models (Huang et al., 2017). SFCN architecture is a lightweight 3D model type originally developed for predicting biological age from brain MRI (see Figure 2 for detailed architecture) (Peng et al., 2021). In terms of parameter counts, the SFCN, ResNet10, ResNet18, and DenseNet121 contained approximately 0.74 million, 3.96 million, 8.30 million, and 11.24 million parameters, respectively.

Model performance was assessed using 5-fold cross-validation, with the fMRI dataset randomly divided into training and validation subsets in an 8:2 ratio. Each trial’s data were assigned entirely to either the training or validation set to reduce the risk of overestimating prediction accuracy. To address the class imbalance in the training datasets, the majority class was randomly undersampled (Batista et al., 2004). All CNN models were implemented in a Python 3.7.4 environment on Ubuntu 18.04, using PyTorch 1.13.1 and CUDA 11.7. Model training was conducted on a single RTX A6000 GPU (NVIDIA, Santa Clara, CA, USA) with 24GB of memory. Key hyperparameters were optimized by using the Bayesian optimization framework Optuna (Akiba et al., 2019). For the three stimulus-based classification tasks, a shared set of hyperparameters was used: learning rate of 1 × 10^−3^, weight decay of 0.4, and batch size of 64. For the two perception-based classification tasks, hyperparameters were separately optimized as follows: for the classification of the Aristotle illusion vs. the Reverse illusion, a learning rate of 3 × 10^−4^, weight decay of 0.4, and batch size of 32 were used; for the classification of occurrence vs. absence of the Reverse illusion, a learning rate of 1 × 10^−5^, weight decay of 0.2, and batch size of 16 were used.

Grad-CAM was employed to generate saliency maps that highlight important regions of the fMRI images contributing to the CNN’s predictions. Grad-CAM produces a saliency map by taking a weighted combination of the feature map activations from the last convolutional layer followed by a rectified linear unit (ReLU) function. The importance weight for each feature map is computed via global pooling of the gradient of the network output with respect to the feature map, as written in Equation 1 (Selvaraju et al., 2017).


(1) $$L_{\text{Grad}-\mathrm{CAM}}=\text{ReLU}(\sum_{k}\alpha_{k}A_{k}),\alpha_{k}=\text{Pool}(\frac{\partial y}{\partial A_{k}})$$

where $A_{k}$ is the *k*th feature map activation, and *y* is the output of the entire network.

### 2.8 Statistical analysis

Two types of statistical analyses were performed in this study. First, repeated-measures ANOVA (rmANOVA) was conducted to examine differences in the illusion rate across the three stimulation conditions: Aristotle, Reverse, and Asynchronous. Perceived distance was defined as the average reported distance between two stimuli for each condition and was also analyzed using rmANOVA to evaluate differences among the three stimulation conditions.

Second, to analyze the saliency maps derived from Grad-CAM, each map was divided into 120 regions of interest (ROIs) based on the Automated Anatomical Labeling (AAL) atlas (Tzourio-Mazoyer et al., 2002). To identify ROIs that consistently exhibited elevated saliency across individuals, we calculated a mean saliency value for each ROI per subject, followed by a grand mean averaged across all ROIs and participants. Individual ROI means were then compared to the grand mean using the Wilcoxon signed-rank test, assuming non-Gaussian distributions. Multiple comparisons were corrected using false discovery rate (FDR) correction. Statistically significant brain regions identified through this analysis were visualized using BrainNet Viewer (Xia et al., 2013). To examine inter-individual variability, subject-wise saliency values were extracted from each statistically significant ROI. For each ROI, saliency values were plotted across all participants, with the group mean and standard error overlaid as summary statistics. A horizontal reference line indicating the grand mean saliency was added to facilitate interpretation.

## 3 Results

### 3.1 Behavioral results

**Figure 3:**
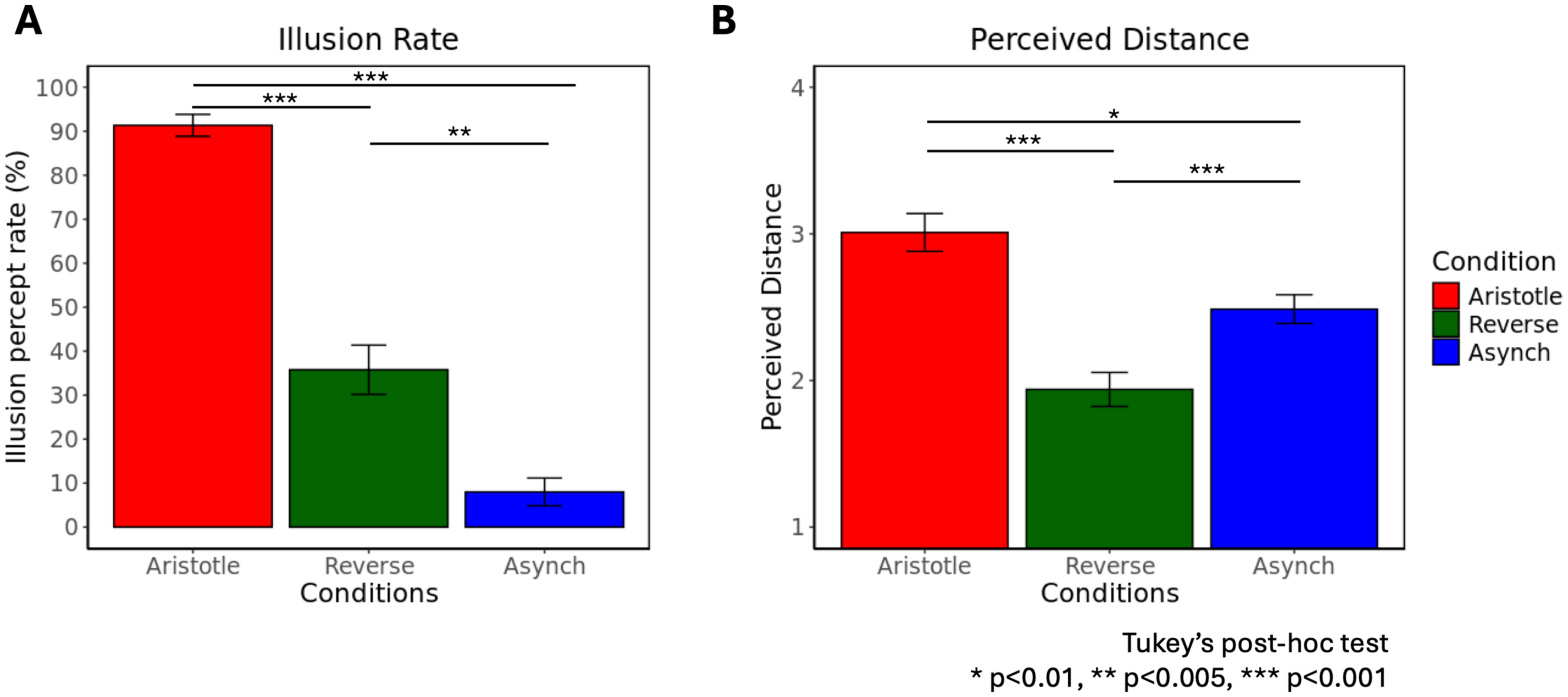
Frequency of illusory percepts and perceived distance across three stimulus conditions: Aristotle, Reverse, and Asynchronous. **(A)** Percentage of illusory percepts across the three stimulus conditions. **(B)** Mean perceived distances across the three stimulus conditions. A score of 1 was assigned when no difference was perceived in the location of stimuli, whereas a score of 4 indicated maximal perceived distance. Means and standard errors are reported.

We observed behavioral results reflecting the illusion effect in both the Aristotle and Reverse conditions, consistent with previous findings (Bufalari et al., 2014). A one-way rmANOVA revealed a significant effect of stimulus condition (Aristotle, Reverse, Asynchronous) on illusion rate [*F*(2,58) = 97.46, *p* = 2.84 $\times 10^{-19}$ ] (Figure 3A). Tukey’s post-hoc test indicated that the illusion rate in the Asynchronous condition was significantly lower than that in both the Aristotle (*p* = 9.59 $\times 10^{-10}$ ) and Reverse conditions (*p* = 1.35 $\times 10^{-3}$ ), corroborating that the Asynchronous stimulus served as a veridical reference. Furthermore, the illusion rate in the Aristotle condition was significantly higher than in the Reverse condition (*p* = 1.21 $\times 10^{-19}$ ), suggesting that the Aristotle stimulus induced a stronger illusion effect.

Another one-way rmANOVA showed a significant effect of stimulation condition on perceived distance [F(2,58) = 24.07, *p* = 2.45 $\times 10^{-8}$ ] (Figure 3B). Tukey’s post-hoc tests revealed that the Aristotle condition led to significantly greater perceived distance compared to both the Reverse (*p* = 2.51 $\times 10^{-6}$ ) and Asynchronous conditions (*p* = 0.01). Moreover, the perceived distance in the Reverse condition was significantly smaller than that in the Asynchronous condition (*p* = 1.98 $\times 10^{-4}$ ). When comparing conditions against the Asynchronous stimulus, the Aristotle condition—despite the actual stimulus being the closest—was perceived as the farthest. In contrast, the Reverse condition—although physically similar in distance to the Asynchronous condition—was perceived as the closest. These results indicate that illusory stimuli (Aristotle and Reverse) induced significant perceptual distortions. Table 1 summarizes participants’ perceptual responses to three tactile stimuli recorded during the fMRI experiments. For the Aristotle and Reverse conditions, illusory and veridical responses are indicated by green and yellow highlights, respectively. These responses served as labels for the fMRI data used in the classification tasks described in section 2.6.

**Table 1:** Behavioral responses for three stimulus conditions: Aristotle (one stimulus), Reverse (two synchronous stimuli), and Asynchronous (two asynchronous stimuli).

Condition	One stimulus perceived	Two stimuli perceived	Non-response
Aristotle	52	544	4
Reverse	212	382	6
Asynchronous	48	542	10

Green-highlighted cells indicate illusory responses, while yellow-highlighted cells represent veridical responses.

### 3.2 Classification of fMRI data

#### 3.2.1 Perception-based classification

Table 2 presents the performances of CNN models in perception-based classification tasks. For the classification between the occurrence of the Aristotle illusion and the Reverse illusion (corresponding to perceiving two vs. one stimulus, respectively), the SFCN achieved the highest performance across all metrics, with an accuracy of 68.0%, precision of 0.67, recall of 0.73, and F1-score of 0.70. Classification accuracy decreased in the following order: SFCN, ResNet10, ResNet18, and DenseNet121. For the classification between the occurrence and absence of Reverse illusion (corresponding to perceiving one vs. two stimuli, respectively), the SFCN again outperformed the other models, achieving an accuracy of 80.1%, precision of 0.74, recall of 0.74, and F1-score of 0.73. ResNet10, ResNet18 and DenseNet121 achieved accuracies of 73.7, 74.0, and 72.9%, respectively. In summary, the number of stimuli perceived by participants was classifiable based on fMRI data with moderate to high accuracy.

**Table 2:** Performance of four CNN models (SFCN, ResNet10, ResNet18, DenseNet121) for two classification tasks based on participants’ perception: (i) occurrence of the Aristotle illusion vs. the Reverse illusion, and (ii) occurrence vs. absence of Reverse illusion.

Model	Occurrence of Aristotle vs. Reverse				Reverse illusion vs. no Reverse illusion			
	Accuracy	Precision	Recall	F1-score	Accuracy	Precision	Recall	F1-score
SFCN	0.684	0.670	0.726	0.695	0.801	0.741	0.735	0.725
ResNet10	0.626	0.609	0.623	0.597	0.756	0.677	0.678	0.653
ResNet18	0.620	0.602	0.649	0.607	0.740	0.645	0.641	0.621
DenseNet121	0.585	0.558	0.564	0.525	0.729	0.634	0.609	0.597

#### 3.2.2 Stimulus-based classification

For all stimulus-based classification tasks (Aristotle vs. Reverse, Reverse vs. Asynchronous, Asynchronous vs. Aristotle), all CNN models yielded maximum accuracies around 0.5 over training epochs, indicating that performance did not improve beyond that of initial random choice (see an example learning curve in the Discussion section). This suggests that the CNN models failed to extract discriminative fMRI image features capable of distinguishing among the three types of applied stimuli.

### 3.3 Grad-CAM analysis

**Figure 4:**
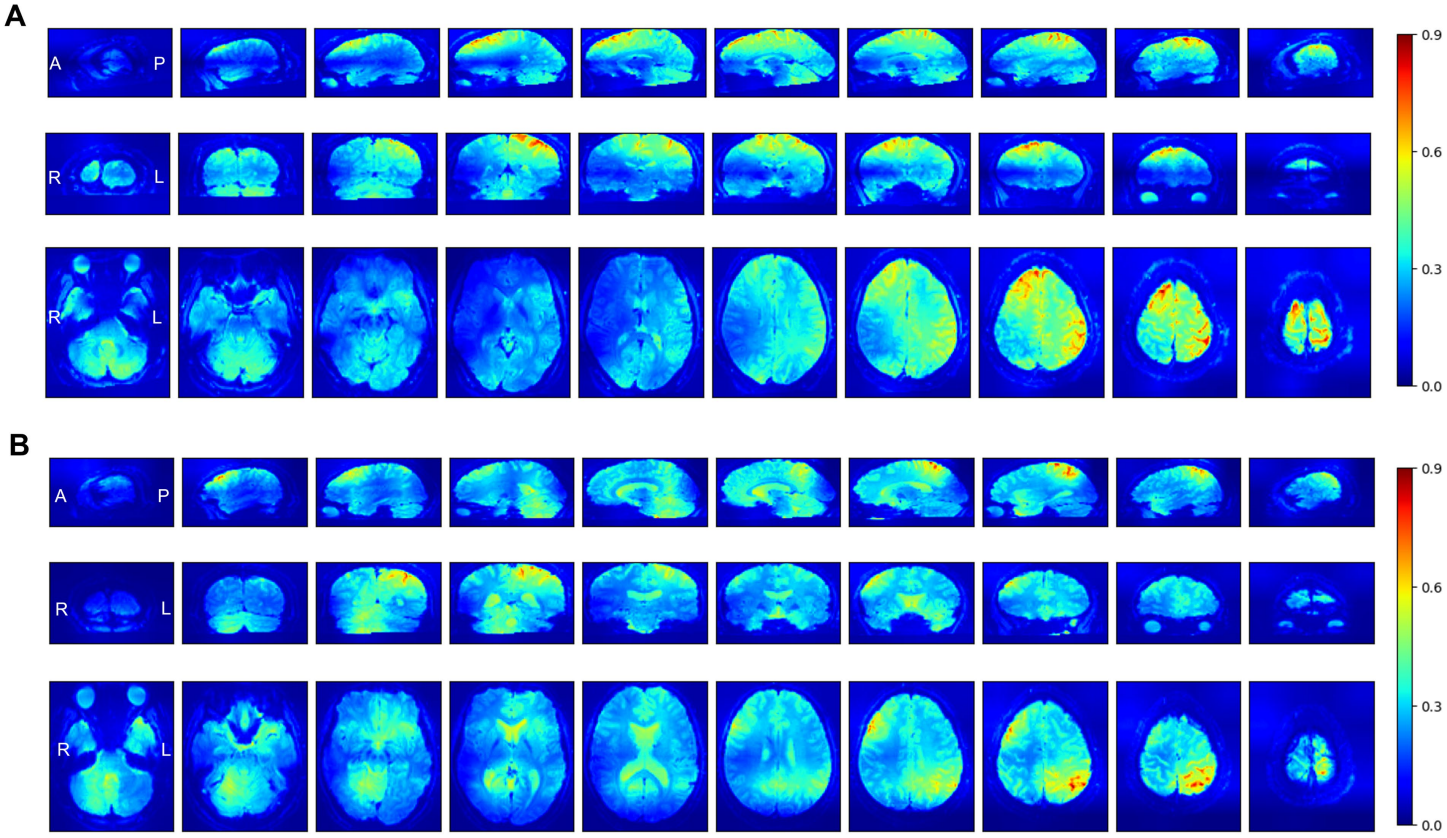
Selected axial, coronal, and sagittal slices of mean Grad-CAM saliency maps from the SFCN model for two perception-based classification tasks: **(A)** Occurrence of the Aristotle illusion vs. Reverse illusion and **(B)** occurrence vs. absence of the Reverse illusion. The displayed maps represent the average of all individual maps in the validation datasets. In the maps, higher intensity values (red) indicate regions to which the SFCN model assigned greater attention during classification.

**Figure 5:**
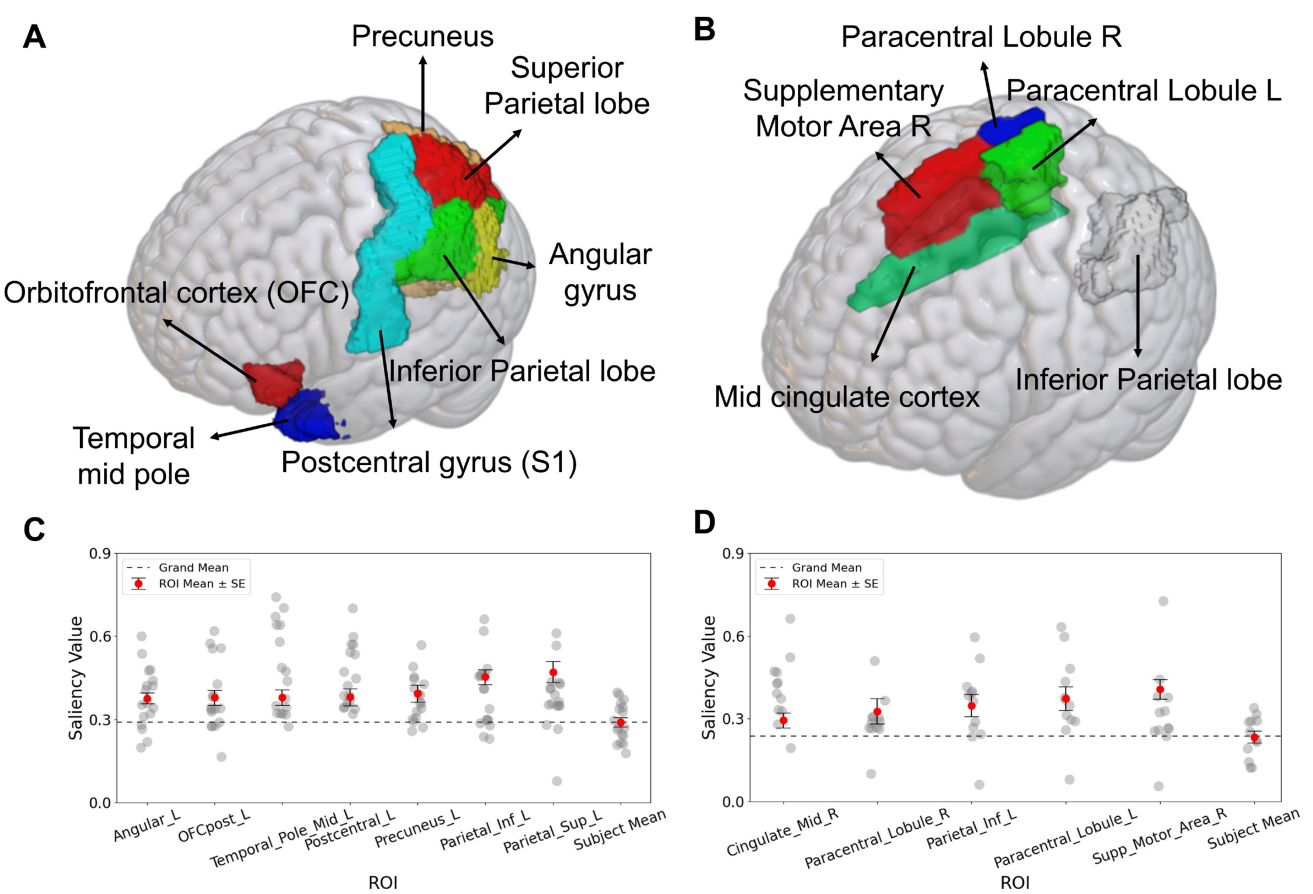
Salient ROIs identified by Grad-CAM analysis for the SFCN classification model. **(A)** For classification of the occurrence of the Aristotle illusion vs. Reverse illusion, the superior parietal lobule, inferior parietal lobule, precuneus, postcentral gyrus (S1), middle temporal pole, orbitofrontal cortex (OFC), and angular gyrus were significantly above the grand mean (*p* < 0.05). **(B)** For the occurrence vs. absence of the Reverse illusion, the supplementary motor area (SMA), paracentral lobule, inferior parietal lobule, and middle cingulate cortex were identified as salient ROIs. **(C)** Subject-wise saliency values for each significant ROI in the Aristotle illusion vs. Reverse illusion. **(D)** Subject-wise saliency values for each significant ROI in the presence vs. absence of the Reverse illusion. Each gray dot represents one participant. Red circles indicate the mean saliency of each ROI, with black bars representing the standard error. The dashed line represents the grand mean across all brain regions.

Grad-CAM was applied to the SFCN model, which exhibited the best performance across the two perception-based classification tasks. Figure 4 presents selected axial, coronal, and sagittal slices of *mean* 3D saliency maps averaged across all participants in each validation set. Note that simple pixel-wise averaging sufficed to obtain the mean saliency map, as all fMRI images had been spatially co-registered during the pre-processing phase. The grand-mean-based ROI analysis of the resulting saliency maps (outlined in Section 2.8) identified significant ROIs for each task as detailed below. For the Aristotle illusion vs. Reverse illusion classification, seven significant ROIs were identified (*p* < 0.05), including the superior parietal lobule (*p* = 0.002), inferior parietal lobule (*p* = 0.001), precuneus (*p* = 0.003), postcentral gyrus (S1) (*p* = 0.003), middle temporal pole (*p* = 0.002), orbitofrontal cortex (OFC) (*p* = 0.003), and angular gyrus (*p* = 0.03). For the occurrence vs. absence of the Reverse illusion, five ROIs were identified, including the supplementary motor area (SMA) (*p* = 0.001), paracentral lobule (*p* = 0.01), inferior parietal lobule (*p* = 0.01), and middle cingulate cortex (*p* = 0.01). Figure 5 shows the identified ROIs for the two tasks using BrainNet Viewer.

Further analysis of inter-individual variability was performed by plotting subject-wise saliency values for each ROI identified as significant in the group-level Grad-CAM analysis (Figures 5C,D). Subject mean values were narrowly distributed and closely aligned with the grand mean, indicating minimal inter-subject variation in baseline saliency levels. In both classification tasks, the ROI-specific saliency values were consistently higher than subject-level means.

## 4 Discussion

This study aimed to identify brain regions involved in the Aristotle illusion using fMRI and deep learning-based decoding. Behavioral results demonstrated that the illusory stimuli effectively induced tactile illusions in participants. To decode the neural representations associated with tactile perception, we trained CNNs for two classification approaches: the first based on the type of applied stimulus (stimulus-based classification) and the second based on the number of perceived stimuli (perception-based classification). The validation results showed that perception-based classification was feasible, achieving moderate to high accuracies, whereas stimulus-based classification was not successful due to ineffective CNN training.

**Figure 6:**
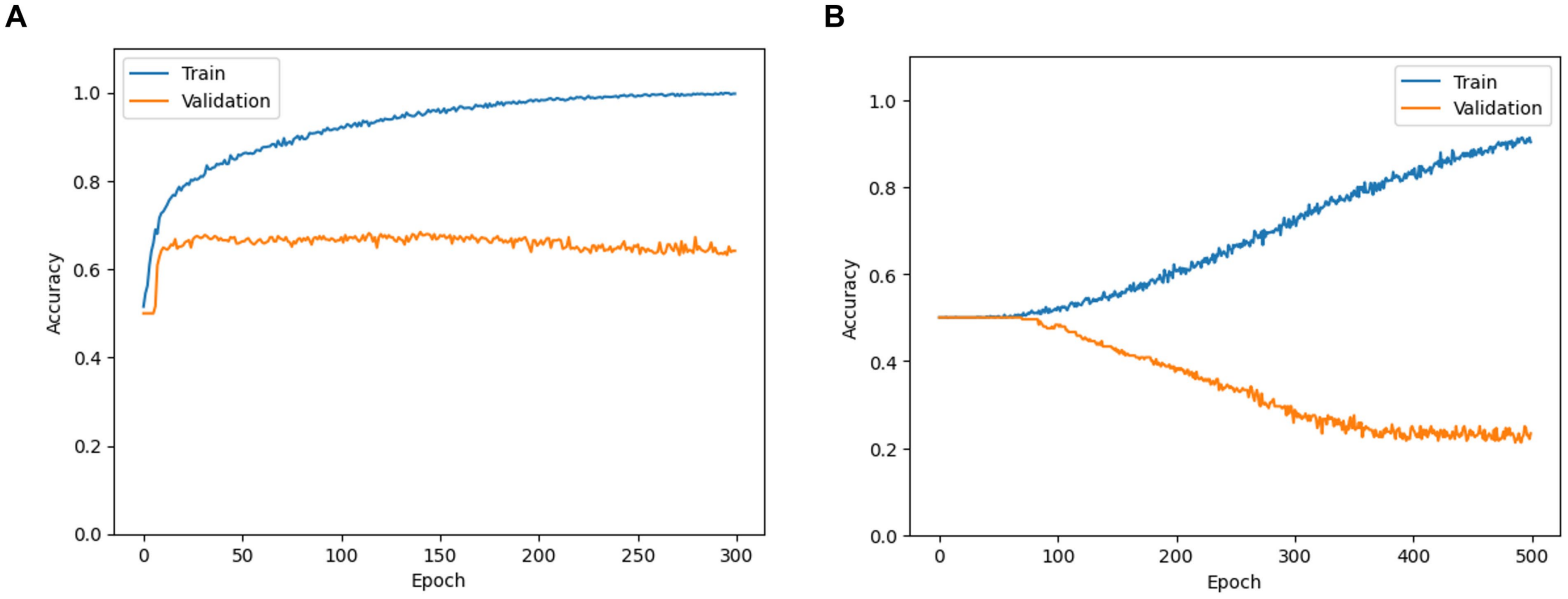
Training and validation accuracy curves obtained while training SFCN models for **(A)** occurrence of Aristotle vs. Reverse (as an example of perception-based classification) and **(B)** Aristotle vs. Asynchronous (as an example of stimulus-based classification).

In the perception-based classification, the CNN models exhibited a typical learning pattern: training accuracy steadily increased over epochs, while validation accuracy initially rose but eventually plateaued or declined (Figure 6A). In the CNN learning for the stimulus-based classification, training accuracy increased over epochs, indicating successful loss minimization. However, validation accuracy decreased from the initial value of approximately 0.5, which resulted from random initialization—suggesting poor model generalization (Figure 6B). These findings imply that fMRI image features may correlate more strongly with participants’ subjective experience than with the type of stimulus applied. Consequently, we applied Grad-CAM analysis solely to perception classification tasks, including the occurrence of the Aristotle illusion vs. the Reverse illusion and the occurrence vs. absence of the Reverse illusion.

The classification between the Aristotle illusion and the Reverse illusion was particularly important for probing distinct neural mechanisms underlying divergent perceptual outcomes. The Grad-CAM-based classification between these two perceptual illusions was therefore aimed at revealing brain regions that not only encode tactile signals but also contribute to higher-level resolution of spatial mismatch between tactile and proprioceptive cues. Identifying these neural substrates provides critical insights into how the brain supports interpretation of mismatched sensory information.

Although the contrast between the occurrence vs. absence of the Aristotle illusion may be theoretically meaningful, it was excluded from the main analysis due to severe class imbalance: 544 trials reflected illusory perception, while only 52 reflected veridical perception. An initial evaluation showed that the model consistently predicted the majority class, resulting in superficially high accuracy but no meaningful discriminative performance. While this task was tested for completeness, the results were not sufficiently informative and are therefore not reported in detail. For the occurrence of the Aristotle vs. Reverse illusion, Grad-CAM analysis identified the parietal regions, primary somatosensory cortex (S1), OFC, and temporal pole as salient brain regions. The key difference between the Aristotle and Reverse illusions lies in how the brain interprets tactile stimuli relative to proprioceptive cues. In both illusions, crossing the fingers creates a mismatch between actual tactile input and the brain’s internal representation of the body. In the Aristotle illusion, a single stimulus is misinterpreted as two due to the altered spatial configuration of the fingers. Conversely, in the Reverse illusion, two separate stimuli are perceived as one because the brain integrates the signals into a single percept. This perceptual difference is reflected in S1 activity. Previous research has implicated S1 with processing the Aristotle illusion (Bufalari et al., 2014). One such study found that the N20 amplitude in S1 was significantly higher under the Reverse illusion condition than under the Aristotle illusion condition, which may account for the robust decoding of the Aristotle vs. Reverse illusions in this region.

Furthermore, we observed a significant difference in the inferior parietal cortex between the two illusions. The parietal region plays an essential role in multisensory integration, involving the proprioceptive and tactile information (Kavounoudias et al., 2008; Blankenburg et al., 2006; Marasco and De Nooij, 2023), suggesting that different types of illusions may be distinctly represented in this area. The OFC, a higher-order brain region known for integrating sensory, motor and associative information (Stalnaker et al., 2015; Wang et al., 2020), has also been implicated in encoding tactile information (Frey et al., 2009). Additionally, the temporal pole is involved in the representation of tactile roughness perception (Kim et al., 2017). These finding suggest that the difference between the Aristotle and Reverse illusions is modulated by both high-level perceptual interpretation and low-level sensory encoding.

When distinguishing the Reverse illusion from non-illusory perception, the goal was to isolate brain regions responsible for integrating conflicting sensory cues and suppressing illusory interpretations under conditions of spatial ambiguity. In this comparison, the tactile stimuli were physically identical, but the perceptual outcomes differed. This contrast allowed us to find brain regions that mediate the failure or success of perceptual disambiguation. Grad-CAM revealed that salient regions in this classification included the IPL, middle cingulate cortex, and SMA. The consistent involvement of the parietal cortex across both classification tasks highlights its key role in tactile illusion processing. This aligns with previous findings showing significantly different P200 responses in the parietal region between illusory and non-illusory trials in the Reverse illusion condition (Bufalari et al., 2014). Our results support the notion that the parietal cortex plays a crucial role in recalibrating sensory information when somatotopic and external spatial reference frames are misaligned. Beyond the IPL, significant activation was also identified in the SMA and middle cingulate cortex. Prior studies have demonstrated that the SMA is involved in encoding tactile roughness (Kim et al., 2017). Moreover, the middle cingulate cortex has been associated with body representation (Popa et al., 2019) and plays a critical role in integrating proprioceptive and sensory signals associated with motor functions (Rahman et al., 2022). Based on these findings, we suggest that middle brain regions also play a role in distinguishing the Reverse illusion.

In addition to group-level findings, we examined inter-individual variability in saliency distributions to evaluate the consistency of model attention across participants. Subject-wise saliency values for each significant ROI were plotted, showing that most participants exhibited elevated saliency relative to the grand mean. This pattern, observed across both classification tasks, suggests that the identified ROIs were not driven by outliers, but rather reflect stable, interpretable patterns across subjects. Furthermore, when comparing these ROI-specific saliency values to each participant’s whole-brain average saliency, we found that the highlighted regions consistently exceeded individual baselines. The narrow distribution of subject-level mean saliency values further supports that the observed patterns were not subject-specific artifacts but reflect anatomically meaningful, region-specific effects. These findings support the robustness of the Grad-CAM results and reinforce the neuroanatomical relevance of the highlighted regions.

Although the classification results were promising, several methodological considerations should be acknowledged to better interpret the findings. First, the relatively small sample size in the fMRI experiments may limit the generalizability of the results. Despite the number of data points for classification was increased through multiple MR acquisitions per subject, the diversity of the data distribution may still have been insufficient for optimal model generalization. This likely explains why the highest accuracy was achieved using the SFCN, which has the smallest number of model parameters among all the models tested. The moderate accuracy in distinguishing between the Aristotle and Reverse illusions may also be attributed to the limited amount of training data. Second, the interpretability of Grad-CAM visualization is closely tied to the classification performance of the models. The explanatory power of the resultant saliency maps may be limited by classification accuracies, particularly in the Aristotle vs. Reverse task, which achieved only 68% accuracy. To improve the reliability of the saliency maps, increasing training data volume and refining vision encoders will be essential to enhance prediction performance.

We adopted a stimulation duration of 15 s to allow participants sufficient time to focus on the tactile input and to obtain a reliable number of fMRI data points per trial. However, it is plausible that the illusion arises within the first few seconds of stimulation, while the prolonged duration may additionally engage higher-order cognitive processes such as sustained attention. To more precisely capture the neural dynamics that occur immediately following the onset of the illusory percept, future studies could employ a faster trial structure or utilize neuroimaging techniques with higher temporal resolution, such as magnetoencephalography, to better understand the real-time integration of proprioceptive and tactile information.

One potential concern involves the possibility that participants had partial awareness of the number of tactile stimuli, which may have influenced cognitive evaluation rather than reflecting purely perceptual experience. However, several aspects of the experimental design were carefully structured to minimize this effect. The stimulus apparatus, although briefly shown to participants, served only to familiarize them with the tactile setup. No pre-training or behavioral sessions were administered prior to scanning, the stimulators remained out of view during the experiment, and the number of stimuli was varied randomly within each session. These precautions ensured that participants could not anticipate or visually confirm the nature of the tactile input, thereby reducing cognitive influences on perceptual responses. Finally, while this study identified local brain regions associated with the tactile illusion, investigating functional connectivity between these regions could yield a deeper understanding of the network-level mechanisms underlying tactile illusions. In future work, we will collect additional fMRI data and employ advanced vision models with enhanced representational capacities, trained on large datasets. We also plan to explore inter-regional connectivity using graph neural networks and related methods (Cai et al., 2022). As we improve classification accuracy, we will update the saliency maps and investigate any changes in brain regions associated with tactile perception.

## 5 Conclusion

To the best of our knowledge, this study was the first to apply deep learning techniques to fMRI data obtained during tactile stimulation to decode the neural correlates of the Aristotle illusion. Our findings demonstrate that perception-driven neural responses distinguish tactile illusions more effectively than stimulus-driven responses. In line with previous research, we confirmed the involvement of the somatosensory cortex and parietal regions in illusion processing through saliency analysis of deep learning models. Additionally, we identified other key regions—such as the OFC, middle temporal pole, SMA, and middle cingulate cortex—as playing significant roles. Future research should focus on refining decoding models, incorporating connectivity analyses, and exploring neuromodulation techniques to further elucidate the mechanisms underlying tactile illusions.

## Data Availability

The datasets presented in this article are not readily available because fMRI images are potentially identifiable. Requests to access the datasets should be directed to Taehoon Shin, shinage@gmail.com.
