# Supplementary figures and images for: Neural decoding of Aristotle tactile illusion using deep learning-based fMRI classification
Source: Front Neurosci. 2025 Jun 19;19:1606801. doi: 10.3389/fnins.2025.1606801 (PMC12222053; doi:10.3389/fnins.2025.1606801)

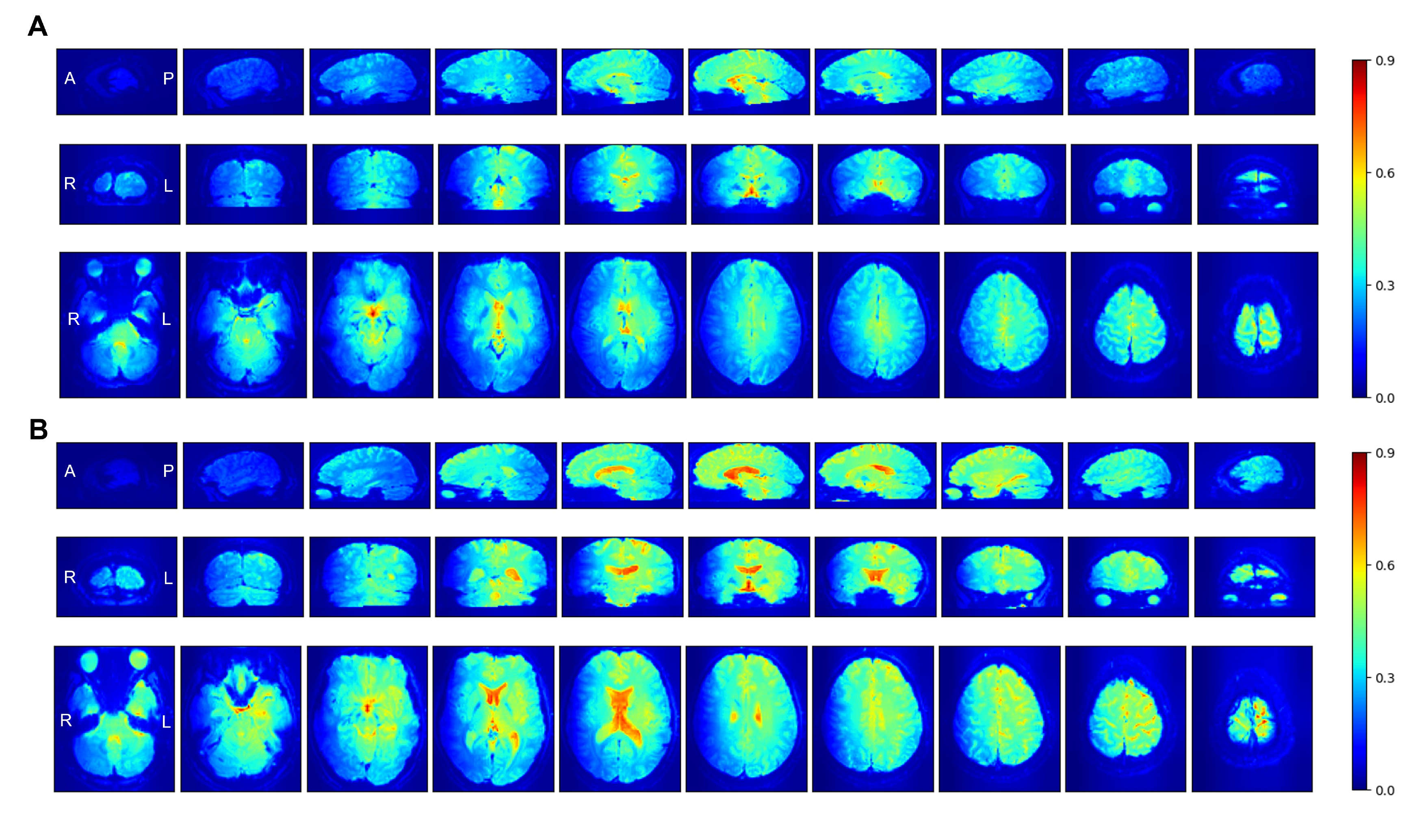

Supplement: Supplementary file 3 [file Image_1.tif]
