# Supplementary material for: Neural decoding of Aristotle tactile illusion using deep learning-based fMRI classification
Source: Front Neurosci. 2025 Jun 19;19:1606801. doi: 10.3389/fnins.2025.1606801 (PMC12222053; doi:10.3389/fnins.2025.1606801)
Supplement: Supplementary file 4 [file Data_Sheet_1.docx]

To evaluate the consistency of Grad-CAM-derived saliency across different CNN architectures, we compared the activation maps generated from ResNet-10 and ResNet-18. Since both models produced highly similar activation patterns across tasks, we report only the results from ResNet-18 for clarity. Full visualizations from both models are provided in Supplementary Figure 1, using the same anatomical layout as Figure 4.

To assess the reliability of the classification performance, we conducted a non-parametric permutation test. Class labels were randomly shuffled and repeated model training 1,000 times. For the occurrence of the Aristotle illusion vs. Reverse illusion, the average accuracy from the permuted models was 53.8%, whereas the actual models achieved 62.6% (ResNet-10) and 62.0% (ResNet-18). For the occurrence vs. absence of the Reverse illusion, the permuted accuracy was 57.2%, while the actual models achieved 73.7% (ResNet-10) and 74.0% (ResNet-18). These results indicate that the observed accuracies exceeded the 95th percentile of the null distribution, suggesting that the performance was significantly higher than would be expected by chance.To further validate the robustness of the saliency results, we examined whether the observed patterns were region-specific rather than subject-specific. Using the same procedure, we tested whether the saliency values in the Grad-CAM-derived ROIs were significantly higher than each subject’s whole-brain average saliency using Wilcoxon signed-rank tests. For the Aristotle illusion vs. Reverse illusion, six out of seven ROIs identified in the SFCN Grad-CAM analysis were replicated in the ResNet-18 Grad-CAM result, including the superior parietal lobule (p = 0.0002), precuneus (p = 0.0002), postcentral gyrus (p = 0.0004), orbitofrontal cortex (p = 0.017), angular gyrus (p = 0.023), and inferior parietal lobule (p = 0.019), with the exception of the middle temporal pole. For the occurrence vs. absence of the Reverse illusion, four out of five previously identified ROIs were replicated, including the supplementary motor area (p = 0.002), paracentral lobule (p = 0.003), middle cingulate cortex (p = 0.004), and precentral gyrus (p = 0.001), with the inferior parietal lobule not observed. These overlaps across tasks support the robustness and anatomical consistency of the saliency patterns derived from Grad-CAM.

Supplementary Figure 1. Selected axial, coronal, and sagittal slices of mean Grad-CAM saliency maps from the ResNet-18 model for two perception-based classification tasks: (A) occurrence of the Aristotle illusion vs. Reverse illusion and (B) occurrence vs. absence of the Reverse illusion. The displayed maps represent the average of all individual maps in the validation datasets. In the maps, higher intensity values (red) indicate regions to which the ResNet-18 model assigned greater attention during classification. ResNet18 exhibited highly similar attention patterns to those of ResNet-10, particularly in the parietal and frontal cortices, supporting the consistency of the saliency results across architectures.

Supplementary Tables 1 and 2 present ROI-wise Wilcoxon test results based on the SFCN, including FDR-corrected p-values across all 120 ROIs. ROIs with FDR-corrected p < 0.05 are visualized in Figure 5.

**Supplementary Table 1.** ROI-wise Wilcoxon signed-rank test results for the occurrence of Aristotle illusion vs. Reverse illusion. Each row corresponds to one ROI, including voxel count, raw p-value, and FDR-adjusted p-value. The full table is available as a separate Excel file.

**Supplementary Table 2.** ROI-wise Wilcoxon signed-rank test results for the occurrence vs. absence of the Reverse illusion. Each row corresponds to one ROI, including voxel count, raw p-value, and FDR-adjusted p-value. The full table is available as a separate Excel file.
